# Physical Activity Intensity Cut-Points for Wrist-Worn GENEActiv in Older Adults

**DOI:** 10.3389/fspor.2020.579278

**Published:** 2021-01-15

**Authors:** François Fraysse, Dannielle Post, Roger Eston, Daiki Kasai, Alex V. Rowlands, Gaynor Parfitt

**Affiliations:** ^1^Alliance for Research in Exercise, Nutrition and Activity (ARENA), University of South Australia, Adelaide, SA, Australia; ^2^National Institute for Health Research Leicester Biomedical Research Centre, Leicester, United Kingdom; ^3^Diabetes Research Centre, University of Leicester, Leicester, United Kingdom

**Keywords:** accelerometer, dominant, non-dominant, sedentary, light, moderate

## Abstract

**Purpose:** This study aims to (1) establish GENEActiv intensity cutpoints in older adults and (2) compare the classification accuracy between dominant (D) or non-dominant (ND) wrist, using both laboratory and free-living data.

**Methods:** Thirty-one older adults participated in the study. They wore a GENEActiv Original on each wrist and performed nine activities of daily living. A portable gas analyzer was used to measure energy expenditure for each task. Testing was performed on two occasions separated by at least 8 days. Some of the same participants (*n* = 13) also wore one device on each wrist during 3 days of free-living. Receiver operating characteristic analysis was performed to establish the optimal cutpoints.

**Results:** For sedentary time, both dominant and non-dominant wrist had excellent classification accuracy (sensitivity 0.99 and 0.97, respectively; specificity 0.91 and 0.86, respectively). For Moderate to Vigorous Physical Activity (MVPA), the non-dominant wrist device had better accuracy (ND sensitivity: 0.90, specificity 0.79; D sensitivity: 0.90, specificity 0.64). The corresponding cutpoints for sedentary-to-light were 255 and 375 g · min (epoch independent: 42.5 and 62.5 mg), and those for the light-to-moderate were 588 and 555 g · min (epoch-independent: 98.0 and 92.5 mg) for the non-dominant and dominant wrist, respectively. For free-living data, the dominant wrist device resulted in significantly more sedentary time and significantly less light and MVPA time compared to the non-dominant wrist.

## Introduction

Population-level measurement of physical activity (PA) and sedentary behaviors (SBs) is important for a number of reasons, including investigating relations with health outcomes (Osborn et al., 2018), quantifying the effect of PA interventions (Mitchell et al., 2019), and establishing secular trends in PA behaviors (Fraysse et al., 2019). Device-measured PA is most commonly carried out using accelerometers worn on the hip, wrist, or thigh. In recent years, most research-grade accelerometers have allowed direct access to raw acceleration data. This allows control over the whole data processing method, resulting in better transparency and reproducibility. Typically, to facilitate classification of activity intensity, acceleration magnitude is collapsed (summed or averaged) into epochs ranging from 1 to 60 s, and thresholds are applied to classify each waking wear epoch as sedentary, light PA (LPA), moderate PA (MPA), or vigorous PA (VPA). These thresholds, also commonly called cutpoints, are established in calibration studies (Evenson et al., 2008; Sasaki et al., 2011; Hildebrand et al., 2017) where acceleration data are recorded concurrently with energy expenditure (EE), obtained from measurement of Vo_2_/CO_2_ using a metabolic cart (Bassett et al., 2012). Some studies have also used other means of estimating EE, such as direct observation, video recordings, or use of the PA compendium. EE is expressed relative to the standard unit of resting metabolism [metabolic equivalent (MET)], and typically, 1.5, 3, and 6 METs are considered the thresholds between sedentary, LPA, MPA, and VPA (Copeland and Esliger, 2009) (although some studies have used 4 and 7 METs for the latter two) (Gorman et al., 2014; Whitcher and Papadopoulos, 2014; Evenson et al., 2015).

These acceleration cutpoints are age-specific and wear-site–specific. The relation between EE and bodily movement changes with age. Physical fitness decreases with age, and as a result, performing the same activity requires higher EE. Because intensity cutpoints are based on fixed values of EE, the corresponding acceleration threshold (reflecting body movement) will tend to decrease the older the target population is. Previous studies have emphasized the need for activity cutpoints specific to older adults (Rejeski et al., 2015; Mankowski et al., 2017). Moreover, while different accelerometer brands generally show excellent agreement in terms of activity classification, they can differ in terms of raw acceleration output (Rowlands et al., 2017). GENEActiv and Axivity devices both use the ADXL345 accelerometer, and their raw acceleration outputs are practically identical, so that it is sensible to use the same cutpoints for both devices. It is not clear whether the same cutpoints can be applied for brands using different accelerometers; for instance, we know that ActiGraph GT9X exhibits overall lower accelerations than GENEActiv and Axivity.

Most cutpoint studies have focused on children or adults, including studies using raw acceleration data from the GENEActiv and ActiGraph GT3x+ devices (Esliger et al., 2011; Phillips et al., 2013; Schaefer et al., 2014; Hildebrand et al., 2017). ActiGraph devices prior to the GT3X provide results not in terms of raw acceleration, but in so-called *counts*, which are filtered signals, with the filtering parameters kept undisclosed by ActiGraph. For the hip-worn ActiGraph GT3X (ActiGraph, Pensacola, FL) accelerometer, ActiGraph *count* cutpoints have been compared in older (66.6 ± 2.9 years) and younger (21 ± 2.5 years) adults, with 824 and 2,207 counts · min^−1^, respectively, associated with moderate-intensity (3 METs) activity (Whitcher and Papadopoulos, 2014). However, few studies have reported cutpoints for older adults (Gorman et al., 2014; Whitcher and Papadopoulos, 2014; Evenson et al., 2015).

For GENEActiv devices, studies by Duncan et al. (2019) and Sanders et al. (2019) have established acceleration cutpoints for older adults (55–77 and 60–86 years old, respectively). Duncan et al. tested the effect of wear site on activity classification, with devices worn on both wrists, waist, and ankle. The activities used were focused on the moderate activity level, and most of the moderate-intensity activities were walking activities, at different speeds. Sanders et al. investigated a non-dominant-wrist-worn GENEActiv and a waist-worn ActiGraph and produced two sets of cutpoints, one optimizing the overall classification accuracy and the other optimizing sedentary sensitivity and MVPA specificity. This study did not compare dominant vs. non-dominant wrist, and we know that acceleration, and therefore cutpoints, can be different between wrists for the same activity (Esliger et al., 2011; Phillips et al., 2013; Duncan et al., 2019). Moreover, both studies placed the emphasis on sedentary and moderate-vigorous intensities, at the expense of light intensity.

Older adults tend to have a lower exercise capacity, spend more time sedentary, and rarely engage in VPA relative to younger adults (Matthews et al., 2008; Troiano et al., 2008; Jefferis et al., 2019). For this reason, an increase in PA could result in improved health. In particular, it has been shown that even LPA is associated with a lower risk of death in the elderly (Ekelund et al., 2019; Klenk and Kerse, 2019). However, most accelerometer-based PA studies tend to focus on the ends of the PA spectrum, that is, sedentary and MVPA. Our study was designed with a focus on light-to-moderate activities, with a goal to achieving better discrimination between sedentary, LPA, and MPA in older adults.

Wrist-worn accelerometers are reported to be more acceptable and better tolerated by children (Fairclough et al., 2016), adolescents (Scott et al., 2017), and adults (Montoye et al., 2020), compared to a hip-worn device, although we do not know whether this is true for older adults for which daily activity patterns and usual clothes are usually very different from younger populations. The identification of PA intensity cutpoints, specific to older adults for wrist-worn accelerometers, and with a stronger focus on the light activity intensities, is warranted. A secondary purpose was to investigate the effect of accelerometer placement [dominant (D) or non-dominant (ND) wrist] on classification accuracy and test–retest reliability, with an emphasis on typical activities in LPA domain—such as grocery shopping, sweeping, washing dishes, gardening, and walking—in order to complement the results of Duncan et al. (2019). We also applied the cutpoints established in this study to a sample of free-living data from the same participants, in order to determine whether there were any differences between devices worn on each wrist.

## Methods

An opportunistic sample of 36 healthy, South Australia community-dwelling older adults was recruited for this study. Inclusion criteria were as follows: older than 70 years, fluent in English, and capable of undertaking general activities of daily living unassisted, such as walking and carrying shopping. Participants' characteristics are presented in Table 1. Twenty-four of the 36 participants were classified as overweight or obese, with a body mass index >25 kg/m^2^. The protocol was approved by the University of South Australia Human Research Ethics Committee. Participants provided written, informed consent.

**Table 1:** Participants' characteristics.

	**Sample size**	**Age (y)**	**Height (cm)**	**Weight (kg)**	**Body mass index (kg/m^**2**^)**
Female	18	76 (4)	158 (6)	66 (11)	26.2 (4.1)
Male	18	78 (6)	174 (5)	86 (11)	28.3 (3.6)

Standard deviations are presented in brackets.

The experiment consisted of two laboratory visits at least a week apart, for test–retest data. The same protocol was repeated for the two visits.

### Laboratory Sessions Protocol

Participants were fitted with one GENEActiv on each wrist (GENEActiv Original, Activinsights, UK). The devices were configured to record data at 100 Hz. Breath-by-breath online gas analysis was conducted via MetaMax 3B (Cortex Biophysik GmbH, Leipzig, Germany) with a face mask (Hans Rudolph Inc., Shawnee, KS, USA). Volume and gas calibration were conducted in accordance with the manufacturer's guidelines prior to each session. Heart rate was measured continuously using a wireless chest-strap telemetry system (RS400; Polar Electro Oy, Espoo, Finland).

They were then asked to perform a series of activities typical of activities of daily living for older adults. These were, in order, as follows:

1. light gardening (digging and removing objects from a sandpit) for 4 min
2. sweeping the floor with a broom while standing for 4 min
3. seated reading for 5 min
4. walking overground at a self-paced comfortable speed for 4 min (a “comfortable, everyday walking pace”)
5. lying in a lateral recumbent position for 5 min
6. washing and drying dishes while standing for 4 min
7. walking overground at a self-paced brisk speed for 4 min (a “brisk pace”)
8. watching TV seated for 5 min, and
9. unpacking groceries while standing for 4 min.

There was a 1-min break between each activity. The researcher wrote down the start and end time of each activity with a 1-s resolution.

### Free-Living Protocol

Between the two laboratory sessions, some participants (*n* = 13) wore a GENEActiv monitor on each wrist for 3 days continuously. They were instructed to keep the devices on at all times as much as feasible, including sleep, and to remove them only for prolonged water immersion. They were also asked to fill in a paper log every day with their bed time and get-up time. This free-living data were processed following the same method as the main study, following which sleep was isolated using self-report logs filled by the participants. Waking wear time was then classified using the cutpoints established in this study. *t*-Tests were performed for the daily average time spent in each of the three intensities in order to check for significant differences between wrists.

### Data Processing

All processing was done in MATLAB (2018b, the MathWorks, Inc.), and the programs are available on request. GENEActiv data were downloaded and low-pass filtered with a cutoff frequency of 20 Hz. The 100-Hz data were collapsed in 5-s epochs by computing the signal vector magnitude (SVM), subtracting gravity, and summing magnitudes over a 5-s window:

$$\begin{array}{l} \text{SV}{\text{M}}_{\text{gs}}=\underset{5s}{\sum }\left|\sqrt{a_{X}^{2}+a_{Y}^{2}+a_{Z}^{2}}-g\right| \end{array}$$

For each activity, the mean, median and standard deviation of the SVM over the central 3 min of activity were computed.

To determine the MET values for each activity, 30-s measured oxygen consumption (mL · kg^−1^ · min^−1^) were averaged for the last 2 min of each activity and divided by 2.8 mL · kg^−1^ · min^−1^ [resting metabolic rate (1 MET) for older adults (Kwan et al., 2004)].

MET data were averaged for each activity. Test–retest reliability analyses were conducted for data collected for each activity across the two testing sessions. The activities were then coded into three categories: sedentary (<1.5 METs), light (1.5–2.99 METs), moderate (3.0–5.99 METs), and vigorous (≥6 METs) (Copeland and Esliger, 2009). The focus of the study was on the light–moderate region of PA, and as such, very few instances of vigorous activity were observed. For the purpose of establishing cutpoints, we therefore opted to group moderate and vigorous activities into a single moderate-to-vigorous (MVPA) level corresponding to METs ≥3.

### ROC Analysis

The goal of the receiver operating characteristic (ROC) curve analysis was to find the cutpoints of accelerometer SVM that most accurately classified each of the three considered activity levels. We followed the same method as Esliger et al. (2011) and Phillips et al. (2013).

The two SVM cutpoints between sedentary and LPA, and between LPA and MVPA, were varied from 1 to 100 g · s (for sedentary to light) and 1–1,000 g · s (for LPA to MVPA) in increments of 1 g · s.

The true- and false-positive, and true- and false-negatives, were then computed, with the definition for true positives as follows:

– Sedentary: if the accelerometer SVM classified the activity as sedentary, and the METs for the activity were <1.5;
– MVPA: if the accelerometer SVM classified the activity as MVPA, and the METs for the activity were at or >3.0.
– Consequently, LPA classification is the one that is above sedentary and below MVPA. A true-positive for LPA classification is therefore as follows: MET values are between 1.5 and 3.0, and acceleration magnitude between the sedentary-to-light and light-to-MVPA thresholds.

Sensitivity and specificity were then computed, and the ROC curves for each of the two cutpoints were created. The optimal cutpoints were defined as the SVM threshold values that maximized the product of sensitivity and specificity. In order to allow comparison with other studies, these cutpoints are presented in two ways:

– scaled to a 60-s epochs equivalent by multiplying the values by 12 (5 s × 12 = 60 s) to allow direct comparison with the cutpoints (g · min) of Esliger et al.;
– averaged over the 5-s epoch length, which makes the resulting cutpoints independent of epoch length.

## Results

### Acceleration SVM as a Function of Activity Intensity

Of the 36 initial participants, five were excluded from analysis: one did not complete all tasks, two withdrew before completion, one had issues with GENEActiv data extraction (identical data for dominant and non-dominant wrists, likely due to operator error during configuration or extraction), and one had mismatches between GENEActiv and Vo_2_ data time stamps. Thus, 31 participants (14 female) were included in the analysis.

The MET values and acceleration SVM for each activity, averaged across participants, are presented in Table 2. Standard deviations and 95% confidence intervals are also presented. There were no significant differences (*p* > 0.05) for either MET values or acceleration between the two time points; we therefore decided to merge the two time points for the subsequent ROC analysis in order to obtain more robust cutpoint estimates. Generally speaking, sitting, lying recumbent, and watching TV were sedentary activities (MET <1.5); light gardening and doing dishes were LPA (1.5 ≤ MET <3.0); and walking and unpacking groceries were MPA (3.0 ≤ MET <6.0) with a few participants performing brisk walking as VPA (MET ≥6.0). Paired *t*-tests resulted in significant differences between acceleration magnitudes (SVM) for the dominant and non-dominant wrists for gardening (*p* < 0.001), sweeping (*p* < 0.001), and doing dishes (*p* < 0.001) for both timepoints, with the dominant wrist exhibiting larger SVM for all these. For all other activities, there were no significant differences at either timepoint.

**Table 2A:** MET and SVM for the nine activities and both timepoints, average (SD) and 95% CI across 31 participants.

		**Gard**		**Sweep**		**Seat**		**WalkS**		**Lie**		**Dishes**		**WalkF**		**TV**		**Grocer**	
		**Mean** **(*SD*)**	**95% CI**	**Mean** **(*SD*)**	**95% CI**	**Mean** **(*SD*)**	**95% CI**	**Mean** **(*SD*)**	**95% CI**	**Mean** **(*SD*)**	**95% CI**	**Mean** **(*SD*)**	**95% CI**	**Mean** **(*SD*)**	**95% CI**	**Mean** **(*SD*)**	**95% CI**	**Mean** **(*SD*)**	**95% CI**
T1	MET	2.79 (0.74)	0.25	3.86 (0.86)	0.29	1.41 (0.24)	0.08	3.68 (0.69)	0.23	1.26 (0.19)	0.07	2.48 (0.40)	0.13	4.94 (1.05)	0.35	1.29 (0.20)	0.07	3.55 (0.61)	0.20
	ND SVM	**26.9** **(13.6)**	4.7	**105.9** **(33.3)**	11.5	12.4 (4.1)	1.4	76.8 (24.4)	8.5	11.9 (12.5)	4.3	**56.8** **(16.4)**	5.7	130.4 (53.3)	18.5	9.3 (3.6)	1.2	55.4 (11.9)	4.1
	D SVM	**80.7** **(26.0)** **<0.001**	9.0	**135.9** **(41.1)** **<0.001**	14.2	14.0 (6.5)	2.3	76.2 (24.7)	8.6	13.8 (15.3)	5.3	**76.5** **(20.2)** **<0.001**	7.0	135.7 (55.4)	19.2	9.6 (5.1)	1.8	60.8 (15.5)	5.4
T2	MET	2.75 (0.58)	0.20	3.85 (0.81)	0.27	1.41 (0.25)	0.08	3.47 (0.91)	0.31	1.23 (0.31)	0.10	2.47 (0.39)	0.13	4.82 (1.33)	0.45	1.27 (0.22)	0.07	3.49 (0.78)	0.26
	ND SVM	**27.7** **(14.4)**	5.0	**96.8** **(31.6)**	10.9	13.9 (4.9)	1.7	70.2 (25.4)	8.8	11.5 (10.8)	3.8	**55.3** **(14.6)**	5.1	131.7 (56.8)	19.7	8.8 (5.1)	1.8	54.6 (11.2)	3.9
	D SVM	**81.1** **(29.9)** **<0.001**	10.4	**127.8** **(36.2)** **<0.001**	12.5	14.5 (4.8)	1.7	71.1 (27.7)	9.6	11.8 (13.0)	4.5	**74.2** **(20.4)** **<0.001**	7.1	135.4 (60.7)	21.0	7.1 (3.3)	1.1	59.9 (13.6)	4.7

Bold SVM values indicate significant differences between dominant (D) and non-dominant wrists (ND). T1, timepoint 1; T2, timepoint 2; gard, light gardening; sweep, sweeping the floor; seat, seated reading; walkS, walking at self-paced comfortable speed; lie, lying in a lateral recumbent position; dishes, washing and drying dishes; walkF, walking at self-paced brisk speed; TV, watching TV seated; grocer, unpacking groceries while standing. Green shading indicates sedentary activities; yellow shading indicates light activities; orange shading indicates moderate activity.

**Figure 1:**
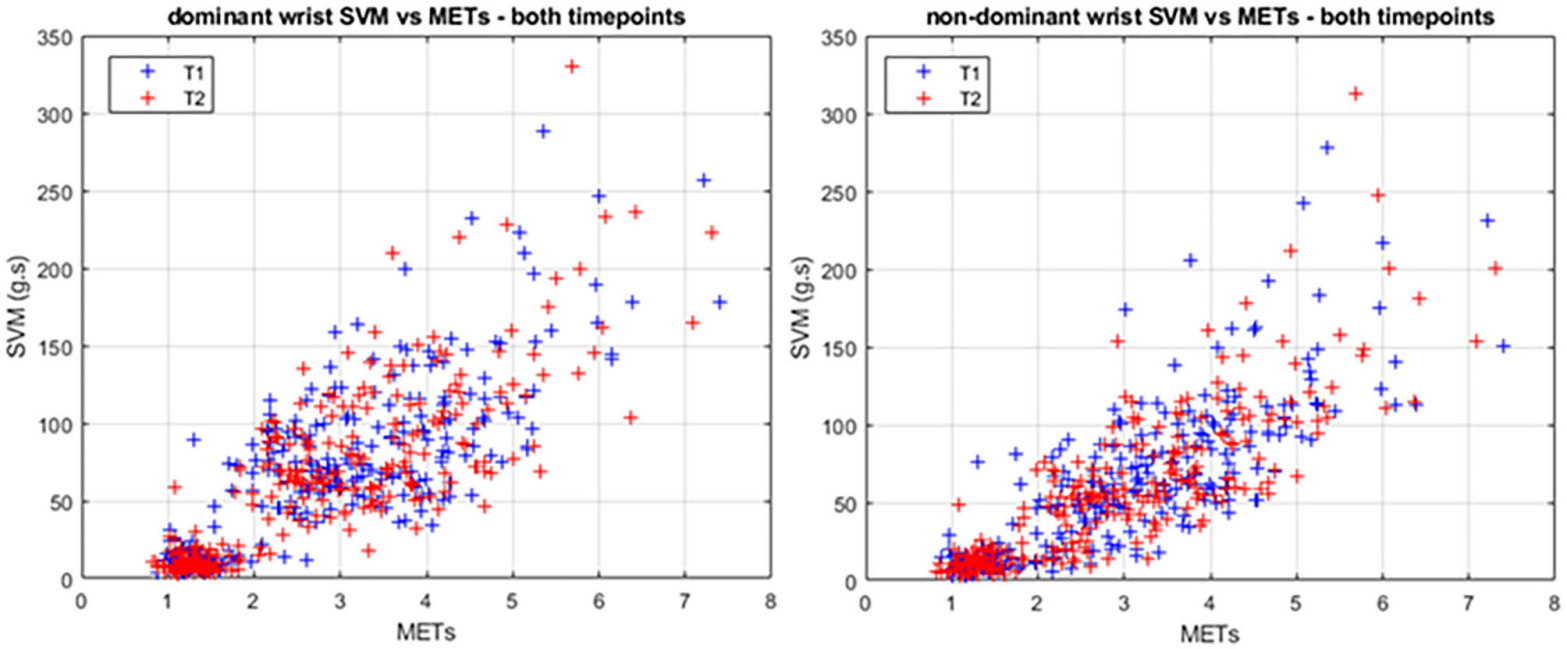
SVM vs. METs for the dominant **(left)** and non-dominant **(right)** wrists. Each point represents one activity and one participant. Timepoint 1 and 2 are shown in blue and red, respectively.

Figure 1 presents the SVM vs. METs for each participant and activity. As can be seen, acceleration SVM increased reasonably linearly with METs. Pearson correlation was *r*^2^ = 0.650 and *r*^2^ = 0.628 for dominant and non-dominant wrists, respectively. Dominant wrist SVM was overall higher than non-dominant; however, the difference was only statistically significant for LPA (1.5 ≤ METs <3.0, *p* < 0.001) and MVPA (METs ≥3.0, *p* < 0.05).

In order to check whether participants had reached steady state for each activity when recording started, we calculated the MET values during the 1-min period preceding the 2 min recording for each activity. As shown in Table 2B, those MET values indicate steady state was reached for each activity. Moreover, data sampled 1 min before each sedentary activity started support the fact that participants had recovered, with MET values of 1.49 (*SD*, 0.34), 1.42 (*SD*, 0.33), and 1.40 (*SD*, 0.33) prior to the three sedentary activities.

**Table 2B:** MET values for the nine activities and both timepoints, average (SD) and 95% CI across 31 participants, compared to the MET values for the same activities, in the minute prior to the recording period.

		**Gard**	**Sweep**	**Seat**	**WalkS**	**Lie**	**Dishes**	**WalkF**	**TV**	**Grocer**
		**Mean (*SD*)**	**Mean (*SD*)**	**Mean (*SD*)**	**Mean (*SD*)**	**Mean (*SD*)**	**Mean (*SD*)**	**Mean (*SD*)**	**Mean (*SD*)**	**Mean (*SD*)**
T1	MET	2.79 (0.74)	3.86 (0.86)	1.41 (0.24)	3.68 (0.69)	1.26 (0.19)	2.48 (0.40)	4.94 (1.05)	1.29 (0.20)	3.55 (0.61)
T2	MET	2.75 (0.58)	3.85 (0.81)	1.41 (0.25)	3.47 (0.91)	1.23 (0.31)	2.47 (0.39)	4.82 (1.33)	1.27 (0.22)	3.49 (0.78)
Minute before recording	MET	2.78 (0.76)	3.78 (0.89)	1.49 (0.34)	3.59 (1.09)	1.42 (0.33)	2.19 (0.44)	4.65 (1.07)	1.40 (0.33)	3.34 (0.75)

### Intensity Cutpoints

**Figure 2:**
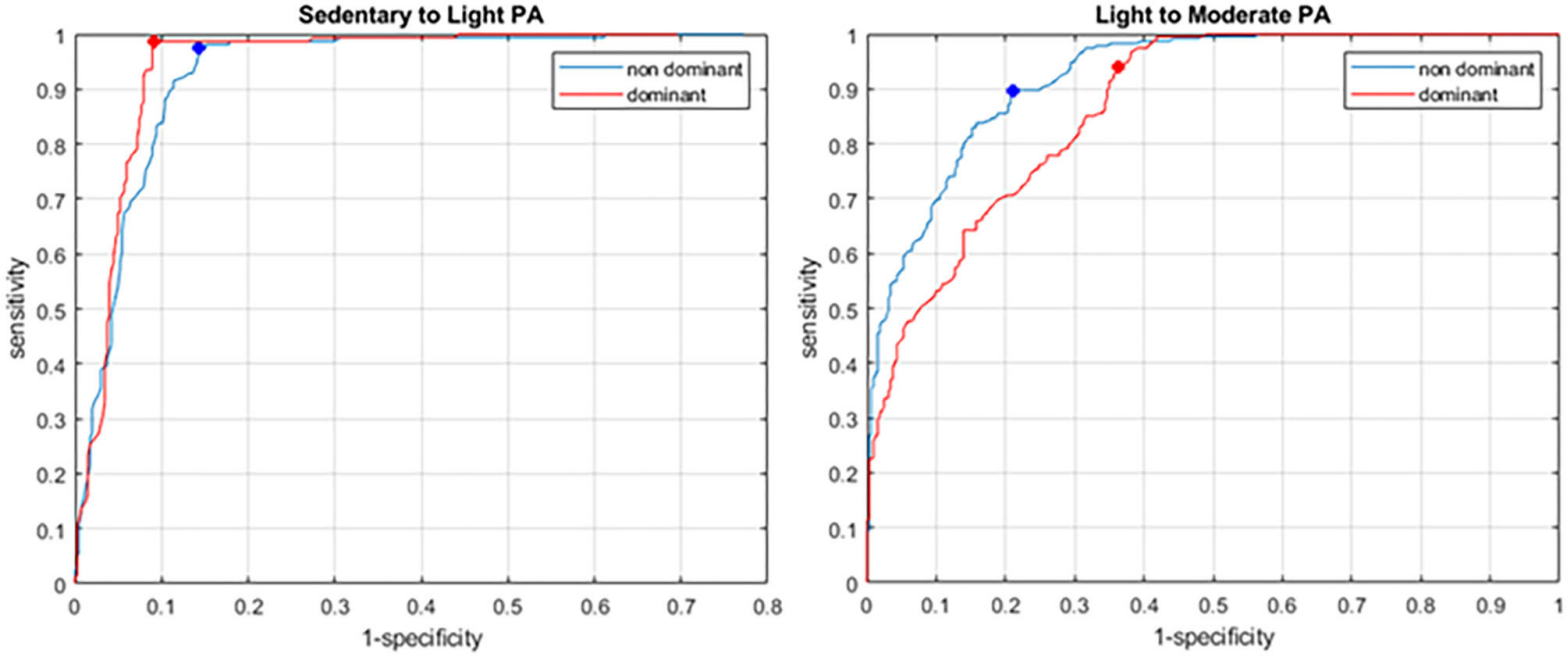
ROC for the sedentary to LPA **(left)** and LPA to MPA **(right)** cut points. Dominant and non-dominant wrists are shown in red and blue, respectively. The dots show the selected cut-points maximizing the product of sensitivity and 1-specificity.

Figure 2 shows the results of the ROC analysis for the sedentary to LPA, and the light to MPA intensity cutpoints. For both wrists, the sedentary to light cutpoint presented excellent sensitivity (0.987 and 0.974 for D and ND, respectively) and relatively lower, although still very acceptable, specificity (0.908 and 0.856, respectively). Corresponding intensity cutpoints for 60-s epoch data were 375 and 255 g · min for the dominant and non-dominant wrists, respectively. The light to MPA cutpoints displayed good sensitivity (0.940 and 0.898 for D and ND, respectively) but lower specificity (0.638 and 0.789, respectively) than the sedentary to LPA cutpoint. Corresponding 60-s intensity cutpoints were 555 and 588 g · min for D and ND wrists, respectively. Table 3 summarizes these findings and presents the results from Esliger et al. (2011), Duncan et al. (2019), and Sanders et al. (2019) for comparison. Age range for our participants was 70–91 years, and that of Esliger et al. was 40–63 years.

**Table 3:** Intensity cutpoints and associated sensitivity and specificity for sedentary to light PA, and light to moderate PA, for the present study and the studies of Esliger et al., Duncan et al., and Sanders et al. cutpoints from both studies were converted to 60-s epoch equivalents at 100 Hz.

**Sedentary to light**	**Cutpoint (g · min)**	**Cutpoint (mg)**	**Sensitivity**	**Specificity**
	**60-s epochs**	**epoch independent**		
**ND—this study**	**255**	**42.5**	**0.97**	**0.86**
ND—Esliger	271	45.2	0.97	0.95
ND—Duncan		19.2	0.74	0.81
ND—Sanders		57	0.43	0.99
**D**—**this study**	**375**	**62.5**	**0.99**	**0.91**
D—Esliger	483	80.5	0.99	0.96
D—Duncan		20.2	0.88	0.84
**Light to moderate**				
**ND—this study**	**588**	**98.0**	**0.90**	**0.79**
ND—Esliger	806	134.3	0.95	0.72
ND—Duncan		89.7	0.67	0.81
ND—Sanders		104	0.81	0.65
**D—this study**	**555**	**92.5**	**0.94**	**0.64**
D—Esliger	550	91.7	1	0.56
D—Duncan		113.8	0.65	0.8

### Free-Living Data

In order to assess the practical differences in intensity estimates between wrists, we used data from a 3-day free-living sample collected on 13 participants that were also part of the main study. Average daily time spent in each intensity level (sedentary, LPA, and MVPA) is presented in Table 4.

**Table 4:** Average daily time spent in each activity intensity for the 3-day free-living data, calculated for the dominant (D) and non-dominant (ND) wrists, using the respective cutpoints.

		***n***	**Mean (min/day)**	**Standard deviation (min/day)**
Sedentary	D	13	630*	75
	ND	13	538*	90
Light PA	D	13	103*	25
	ND	13	209*	57
MVPA	D	13	141	50
	ND	13	134	44

Asterisks indicate significant differences (p <0.05) between dominant and non-dominant wrists.

The dominant wrist and associated cutpoints resulted in significantly more (*p* < 0.01) sedentary time (+92 min/day average), and conversely, significantly less LPA time (−106 min/day average). There was no significant difference (*p* = 0.42) for MVPA time.

## Discussion

This study fills a gap in the literature regarding intensity cutpoints for older adults when using the GENEActiv accelerometer, with an emphasis on the LPA and MPA intensity. Additionally, it examines the benefits of wearing the accelerometer on the dominant or non-dominant wrist in terms of activity intensity classification accuracy.

There are a number of published cutpoints for the GENEActiv on adult populations (Esliger et al., 2011; Hildebrand et al., 2017; Duncan et al., 2019; Sanders et al., 2019). However, Hildebrand et al. (2017) used a different method of data processing, in that they rounded the negative acceleration values to zero rather than taking the absolute value. Therefore, our results can only be compared with those of Esliger et al. (2011). In this regard, our cutpoints are generally lower than those of Esliger et al. (2011) for both sedentary to LPA and LPA to MPA, with the exception of the LPA to MPA cutpoint for the dominant wrist. In comparison to Esliger et al. (2011), the corresponding intensity cutpoints for sedentary-to-light were 6% lower for the non-dominant (255 vs. 271 g · min) and 22% lower for the dominant wrist (375 vs. 483 g · min), and the light-to-moderate cutpoint was 27% lower on the non-dominant wrist (588 vs. 806 g · min), but similar for the dominant wrist (555 vs. 550 g · min). The population of Esliger et al. (2011) was younger than ours, so these findings are in line with the fact that cutpoints become lower for the same intensity with increasing age. This is most notable when comparing children and adult cutpoints, and our study indicates that this trend continues between adults and elderly, in agreement with the findings of Whitcher and Papadopoulos (2014).

Usually, accelerometers are worn on the non-dominant wrist, as it is believed to be a better estimate of overall body movement and therefore activity intensity. In particular, sedentary activities involving mostly dominant arm movement, such as writing, eating, or smoking, could result in the activity being erroneously classified at a higher intensity than it really is. Our ROC results indicate that classification accuracy is comparable for sedentary to LPA, with very high sensitivity and specificity for both dominant and non-dominant wrists, and only slightly lower specificity for the dominant wrist. Note, however, that in this case the specificity is >0.9, which indicates a very good accuracy. Esliger et al. (2011) report similar sensitivities but higher specificities; this may be due to the fact that our study has a continuous range of METs and acceleration SVM (Figure 2), whereas the activities used in the study of Esliger et al. (2011) resulted in a more clustered distribution, which may have contributed to higher specificity (fewer false-positives). Finally, posture (standing vs. sitting) was not considered in the reference method; therefore, the sedentary-to-light cutpoint may be misclassifying light activities as sedentary, if the person is standing up but not moving his/her arms much. Additionally, we used a base value for resting METs for all participants instead of measuring each participant's base resting METs individually, which may have affected the final cutpoint values at all levels.

For the LPA to MPA cutpoint, classification accuracy drops for both wrists; in particular, the specificity is much lower, at 0.638 and 0.789 for dominant and non-dominant wrists, respectively. This was also observed in the results of Esliger et al. (2011). Lower specificity indicates more false-positives, i.e., the accelerometer classified the activity as MPA (or VPA), whereas the activity was actually lower than MPA according to MET values. One reason for this may be that some LPA-level activities involve large or rapid arm movement, while overall the body is standing still: washing dishes and light gardening would be examples from our study. This would also explain the fact that the non-dominant wrist device showed better specificity (0.789) than the dominant one (0.638); as mentioned above, some LPAs primarily involve dominant arm movement; therefore, the risk of misclassifying these as MPA is higher with a dominant wrist device, resulting in more false-positives. Table 2 confirms this: our LPA-level activities showed significantly higher accelerations of the dominant wrist compared to the non-dominant.

Cutpoints established by Duncan et al. (2019) and Sanders et al. (2019) are more difficult to compare directly to ours, because these studies round negative magnitudes to 0 rather than take their absolute value, which causes overall acceleration magnitudes to be lower and therefore cutpoints to be lower. Nevertheless, it can be seen that the sedentary-to-light cutpoints from Duncan et al. are lower than ours by a factor of 2 and 3 for the non-dominant and dominant wrists, respectively (and Duncan having very similar cutpoints for both wrists). The Sanders cutpoints reported in Table 2 are those established using their so-called “Se” method, which aims at maximizing detection of sedentary and MVPA levels, at the expense of LPA. The Sanders cutpoint for sedentary-to-light, at the non-dominant wrist, is 57 mg, approximately 27% higher than ours, which is consistent with their methodology of minimizing false-negatives for sedentary time (and therefore results in a higher cutpoint value).

The target population for this study was older adults, who generally have relatively high levels of sedentary time (Matthews et al., 2008). In this population, a shift in PA from sedentary to light is expected to have positive health outcomes (Stamatakis et al., 2018; Jefferis et al., 2019). In that regard, having a better discrimination between sedentary and LPA should be seen as positive as it allows a finer detection of improvements in PA behaviors. Note that the UK Biobank study used the dominant wrist (Doherty et al., 2017), whereas the majority of other large-scale studies have used the non-dominant wrist [e.g., NHANES (Matthews et al., 2008), LSAC Checkpoint (Fraysse et al., 2019)].

Our study seems to indicate that a dominant-wrist-worn device achieves a better discrimination between sedentary and LPA intensities. However, the difference between dominant and non-dominant wrists remains small, and the present study is by design limited to a small number of laboratory-based activities. Of note, the sedentary-level activities we tested involve about equal movement of the right and left hands (lying, seated reading, and watching TV), whereas our light-intensity activities (gardening and washing dishes) involve mostly the dominant hand. This could have caused the difference in classification we see here between the two devices.

Similarly, for the light-to-moderate threshold, walking is not associated with large differences between dominant and non-dominant accelerations, whereas activities such as gardening are. The proportion of activities that are predominantly performed with the dominant hand will determine the magnitude of this effect. Comparing acceleration magnitudes from both wrists using free-living data has provided more insight into this issue with a younger population (Rowlands et al., 2019), but remains to be done with data for an older population. Cross-validation of the cutpoints using an independent sample would provide better ecological validity to these findings. Additionally, reallocation of LPA into MPA or VPA also has positive effects, and in this case, the non-dominant wrist provides better discrimination. Overall, it is still unclear which of the dominant or non-dominant wrist provides better estimates of activity intensities. Finally, a recent study indicates that temporal patterns of PA are associated with health outcomes in older adults (Li et al., 2019); in this regard, obtaining cutpoints that allow good separation of PA levels is even more critical.

When the dominant and non-dominant cutpoints were applied to free-living data, results showed that the dominant wrist resulted in significantly more time spent sedentary, at the expense of time spent in LPA (Table 3). This result is expected considering the higher sedentary-to-light cutpoint for the dominant wrist. It is, however, unclear which wrist is a better estimate of sedentary and LPA intensities. One possibility is that the laboratory study exacerbated differences between wrists that are not as large in free-living. A recent study by Migueles et al. (2019) found indeed that the dominant wrist exhibited overall larger acceleration magnitudes; however, the resulting difference they found in cutpoints (50 and 45 mg for dominant and non-dominant, respectively) is smaller than the one we found here, suggesting the possibility that our laboratory activities favored motion of the dominant arm. It is also worth noting the large difference in sedentary-to-light cutpoints found by Sanders et al. (2019) when optimizing for either overall best intensity discrimination (20 mg) compared to optimizing for sedentary detection (57 mg).

On a side note, the MET values for the comfortable and brisk walks were significantly different (*p* < 0.001), and both were in the range of MPA (>3.0 METs). While current guidelines advocate walking at a “brisk” pace for health benefits (American College of Sports Medicine, 2017), our data indicate that a self-selected “comfortable” walking speed should be enough in this population, with associated MET values >3.0. Instructions to participants to “walk at a comfortable pace” indeed resulted in moderate-intensity activity according to MET recordings. Moreover, the fact that comfortable and brisk paces were significantly different in terms of energy spent indicates that using self-selected walking speeds in a laboratory study is a feasible method, and more ecologically valid than treadmill walking.

In summary, we provide modified cutpoints for sedentary, LPA, and MVPA in older adults. However, the question of accelerometer wear site (dominant or non-dominant wrist) still remains. In particular, if studying a relatively sedentary population for which most of the PA will be LPA, such as the retired older adults in our study, it may be beneficial to use dominant-wrist-worn devices as our data suggest they provide more accurate estimates of time spent in LPA vs. MPA.

## Data Availability Statement

The raw data supporting the conclusions of this article will be made available by the authors, without undue reservation.

## Ethics Statement

The studies involving human participants were reviewed and approved by University of South Australia Human Research Ethics Committee. The patients/participants provided their written informed consent to participate in this study.

## Author Contributions

GP, FF, and DP conceptualized the study. DP and DK led the data collection. FF, DP, and DK analyzed the data. FF led the writing. GP, AR, and RE provided expert advice and critical review of this manuscript. All authors reviewed the manuscript.

## Conflict of Interest

The authors declare that the research was conducted in the absence of any commercial or financial relationships that could be construed as a potential conflict of interest.
